# Cancer cells differentially modulate mitochondrial respiration to alter redox state and enable biomass synthesis in nutrient-limited environments

**DOI:** 10.1101/2025.05.09.653205

**Published:** 2025-05-10

**Authors:** Sarah M. Chang, Muhammad Bin Munim, Sonia E. Trojan, Anna Shevzov-Zebrun, Keene L. Abbott, Matthew G. Vander Heiden

**Affiliations:** 1 Koch Institute for Integrative Cancer Research, Massachusetts Institute of Technology, Cambridge, MA, USA; 2 Department of Biology, Massachusetts Institute of Technology, Cambridge, MA, USA; 3 Jagiellonian University Medical College, Faculty of Medicine, Chair of Medical Biochemistry, Krakow, Poland; 4 Dana-Farber Cancer Institute, Boston, MA, USA

## Abstract

The cell NAD+/NADH ratio can constrain biomass synthesis and influence proliferation in nutrient-limited environments. However, which cell processes regulate the NAD+/NADH ratio is not known. Here, we find that some cancer cells elevate the NAD+/NADH ratio in response to serine deprivation by increasing mitochondrial respiration. Cancer cells that elevate mitochondrial respiration have higher serine production and proliferation in serine limiting conditions than cells with no mitochondrial respiration response, independent of serine synthesis enzyme expression. Increases in mitochondrial respiration and the NAD+/NADH ratio promote serine synthesis regardless of whether serine is environmentally limiting. Lipid deprivation can also increase the NAD+/NADH ratio via mitochondrial respiration in some cells, including cells that do not increase respiration following serine deprivation. Thus, in cancer cells where lipid depletion raises the NAD+/NADH ratio, proliferation in serine depleted environments improves when lipids are also depleted. Taken together, these data suggest that changes in mitochondrial respiration in response to nutrient deprivation can influence the NAD+/NADH ratio in a cell-specific manner to impact oxidative biomass synthesis and proliferation. Given the complexity of tumor microenvironments, this work provides a metabolic framework for understanding how levels of more than one environmental nutrient affects cancer cell proliferation.

## Introduction

Rapidly proliferating cells, including cancer cells, must acquire biomass precursors such as amino acids, lipids, and nucleotides to support cell growth and division (Deberardinis, 2008; Faubert, 2020; Hanahan, 2011; Pavlova, 2017; Vander Heiden, 2017). While cells can obtain biomass precursors from the environment, many tissue and tumor environments can be deficient in necessary nutrients (Cognet, 2024; Gullino, 1964; Lyssiotis, 2017). In these conditions, cells depend on biosynthesis to accumulate sufficient biomass for proliferation (Apiz Saab, 2023; Ferraro, 2021; Ngo, 2020; Sciacovelli, 2022; Sullivan, 2019b; Sullivan, 2019a). Synthesis of many biomass precursors involve oxidation reactions, which require the redox cofactor NAD+ to act as an electron acceptor. There is accumulating evidence that NAD+ availability and the cell redox state (NAD+/NADH ratio) can limit the synthesis of biomass precursors, including aspartate (Birsoy, 2015; Sullivan, 2015), asparagine (Krall, 2021), fatty acids (Li, 2022), serine (Baksh, 2020; Bao, 2016; Broeks, 2023; Diehl, 2019), and nucleotides (Bao, 2016; Diehl, 2019; Wu, 2024). Thus, the NAD+/NADH ratio impacts cancer cell proliferation in nutrient environments that increase the dependence on biosynthetic oxidation reactions. However, what determines the cellular NAD+/NADH ratio and whether the NAD+/NADH ratio is modulated in response to environmental nutrient availability is not well characterized. Moreover, whether differences in the NAD+/NADH ratio across cell types affect sensitivity to different nutrient limitations is not known.

Some tumor microenvironments have low levels of serine, an amino acid used to produce many biomass components, including proteins, nucleotides, glutathione, and lipids. Thus, low serine environments can limit cancer cell proliferation and tumor growth (Banh, 2020; Maddocks, 2013; 2017; Ngo, 2020; Sullivan, 2019b). In serine depleted environments, cancer cells must increase serine synthesis to maintain proliferation (DeNicola, 2015; Labuschagne, 2014; Locasale, 2011; Possemato, 2011; Sullivan, 2019b). Serine synthesis involves the conversion of the glycolytic intermediate 3-phosphoglycerate (3-PG) into serine. The production of 3-PG from glucose involves an oxidation reaction catalyzed by glyceraldehyde 3-phosphate dehydrogenase (GADPH). 3-PG is then oxidized by phosphoglycerate dehydrogenase (PHGDH) in the first step of the serine synthesis pathway. Both GADPH and PHGDH require NAD+ as an electron acceptor to enable serine synthesis. Experimentally suppressing NAD+ regeneration from NADH decreases serine synthesis, which can be rescued by oxidizing the NAD+/NADH ratio via exogenous electron acceptor supplementation (Bao, 2016; Diehl, 2019; Gravel, 2014). Similarly, *de novo* fatty acid synthesis can be constrained by the NAD+/NADH ratio, as the production of fatty acids involves multiple oxidation reactions to generate citrate from either glucose or glutamine. Thus, decreasing the NAD+/NADH ratio sensitizes cells to environmental lipid depletion (Li, 2022). Together, these findings demonstrate that exogenous changes to the NAD+/NADH ratio can alter serine and citrate production and impact proliferation in serine or lipid-depleted environments. However, auxotrophy for a single nutrient is not sufficient to determine whether cancer cells can form tumors in a tissue, where levels of multiple nutrients may be environmentally limiting for proliferation (Abbott, 2024; Sivanand, 2024; Sullivan, 2019a). Thus, we aimed to uncover how the endogenous NAD+/NADH ratio of cancer cells is regulated to influence biosynthesis in different nutrient depleted environments and its role in determining cancer cell proliferation when multiple oxidized nutrients are limiting.

By culturing cancer cells in serine depleted conditions and examining the relationship between the NAD+/NADH ratio and serine synthesis, we find that the NAD+/NADH ratio is elevated in response to serine deprivation in some but not all cancer cells. The elevated NAD+/NADH ratio is driven by increased mitochondrial respiration and promotes serine synthesis to confer resistance to environmental serine depletion. A similar relationship between mitochondrial respiration and the NAD+/NADH ratio was observed in lipid-depleted environments, where higher mitochondrial respiration in some cells led to elevated NAD+/NADH ratios and enhanced citrate synthesis. Lastly, we find that any perturbation that increases the NAD+/NADH ratio, including either serine or lipid deprivation, led to both elevated serine and citrate production, regardless of what nutrient was depleted from the environment. Together, these data reveal that the NAD+/NADH ratio is regulated by mitochondrial respiration in response to environmental nutrients in a cancer cell-specific manner and that this influences oxidative biosynthesis and proliferation. These data also provide insight into how levels of multiple nutrients in tissue microenvironments may cooperate to enable biomass synthesis and cell proliferation. This study is based in part on work performed for a PhD dissertation (Chang, 2024).

## Results

### Modulating the cell NAD+/NADH ratio proportionally alters serine synthesis

**Figure 1.**
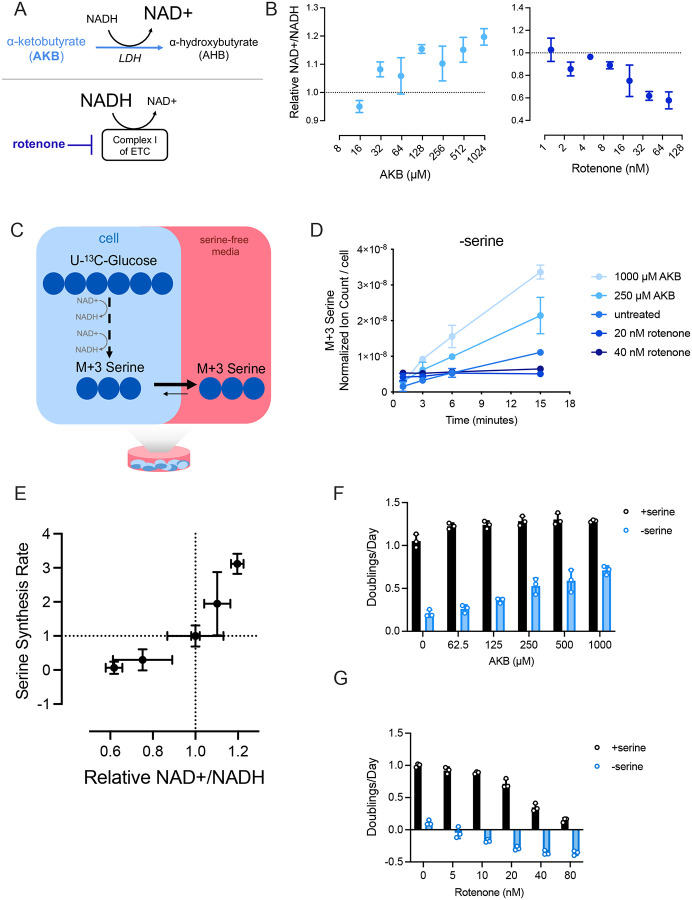
The NAD+/NADH ratio is proportional to serine synthesis rate. **(A)** α-ketobutyrate (AKB) is an exogenous electron acceptor that promotes the oxidation of NADH into NAD+ through its conversion to α-hydroxybutyrate (AHB). Rotenone inhibits activity of complex I of the mitochondrial electron transport chain (ETC) to block NADH oxidation into NAD+. **(B)** NAD+/NADH ratio measured in A549 cells cultured without serine and indicated concentrations of AKB (left) and rotenone (right) for 24 hours. NAD+/NADH ratios are normalized to untreated cells cultured without serine, n=3. **(C)** Schematic depicting NAD+ requirement to produce serine labeled (M+3) from U-^13^C-glucose. Under culture conditions without serine, intracellular serine rapidly effluxes from cells into culture media. Thus, cells and media were jointly analyzed at each time point of kinetic tracing to better capture serine levels. **(D)** Serine labeled (M+3) from U-^13^C-glucose over time in A549 cells cultured without serine and with indicated concentrations of AKB and rotenone for 24 hours prior to U-^13^C-glucose exposure. Serine levels are normalized to internal norvaline standard and cell number measured in indicated conditions, n=3. **(E)** Relative NAD+/NADH ratios from A549 cells cultured without serine after exposure to 250 μM AKB, 1000 μM AKB, 20 nM rotenone, or 40 nM rotenone for 24 hours are plotted against the serine synthesis rates from corresponding conditions as shown in D. Serine synthesis rates are calculated from labeled serine (M+3) after 1 and 15 minutes of U-^13^C-glucose exposure and normalized to untreated and serine-free culture conditions, n=3. **(F)** Proliferation rate (doublings per day) of A549 cells cultured with and without serine and the indicated concentration of AKB for 72 hours, n=3. **(G)** Proliferation rate (doublings per day) of A549 cells cultured with and without serine and the indicated concentration of rotenone for 72 hours, n=3. Data shown represent means ± SD.

To begin investigating the processes that determine the cell NAD+/NADH ratio and influence biomass synthesis, we examined the relationship between the NAD+/NADH ratio and serine synthesis in serine replete and serine limited conditions. Previous studies have found that decreasing the cell NAD+/NADH ratio (more reduced) can suppress serine production (Baksh, 2020; Bao, 2016; Broeks, 2023; Diehl, 2019). To confirm this, we varied the cell NAD+/NADH ratio upon serine withdrawal and measured the serine synthesis rate of A549 non-small cell lung cancer cells, which transcriptionally upregulate the serine synthesis enzyme PHGDH (DeNicola, 2015). We increased the NAD+/NADH ratio by treating cells with the exogenous electron acceptor alpha-ketobutyrate (AKB), which can be reduced to alpha-hydroxybutyrate to regenerate NAD+ (Sullivan, 2015) (Figure 1A). We decreased the NAD+/NADH ratio by treating cells with rotenone, an inhibitor of mitochondrial complex I, which regenerates NAD+ in support of respiration. AKB and rotenone dose-dependently increased and decreased the cell NAD+/NADH ratio, respectively (Figure 1B). To measure the serine synthesis rate in cells, we performed kinetic isotope tracing using uniformly ^13^C-labeled glucose (U-^13^C-glucose) and measured production of M+3 labeled serine (Figure 1C). Because intracellular serine, including newly synthesized serine, rapidly exchanges with the serine-free extracellular environment (Labuschagne, 2014), we collected both media and cells to ensure detection of all newly synthesized serine produced from glucose. We find that increasing the cell NAD+/NADH ratio with AKB led to proportionally higher serine synthesis rates whereas lowering the cellular NAD+/NADH ratio with rotenone led to proportionally lower serine synthesis rates (Figure 1D). This was not explained by changes in PHGDH protein expression, as altering the NAD+/NADH ratio with AKB or rotenone did not affect PHGDH protein levels despite modulating serine synthesis (**Supplementary Figure 1A,B**). Of note, we find that serine synthesis rate is positively correlated with the cell NAD+/NADH ratio (Figure 1E). Accordingly, the NAD+/NADH ratio is also positively correlated with proliferation rate following serine depletion (Figure 1F,G; **Supplementary Figure 1C**). Together, these data demonstrate that modulating the NAD+/NADH ratio leads to proportional changes in serine synthesis rates and proliferation in serine-depleted environments.

### Cancer cells with elevated NAD+/NADH ratios following serine withdrawal exhibit greater serine synthesis

**Figure 2.**
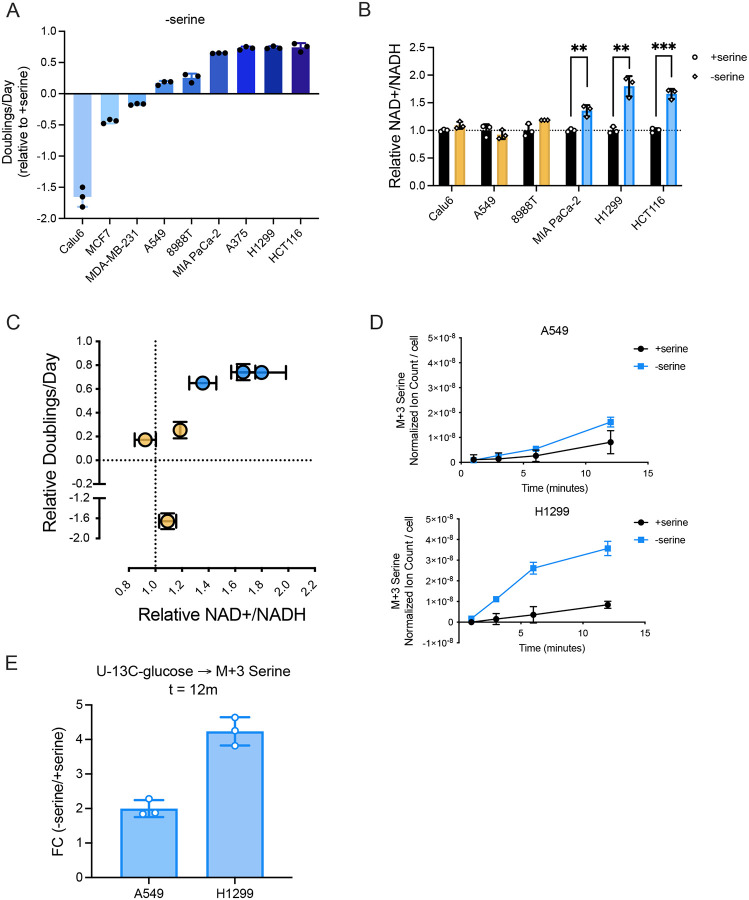
The NAD+/NADH ratio differs between cancer cells upon serine withdrawal and correlates with proliferation in serine-depleted conditions. **(A)** Proliferation rate (doublings per day) of indicated cancer cells cultured without serine for 72 hours normalized to proliferation rate of corresponding cancer cells cultured with serine, n=3. **(B)** NAD+/NADH ratios of indicated cells cultured with and without serine for 24 hours. Yellow indicates cells with unaltered NAD+/NADH ratios and blue indicates cells with elevated NAD+/NADH ratios when cultured without serine. Values are normalized to the NAD+/NADH ratios of cells cultured with serine, n=3. **(C)** Relative NAD+/NADH ratios of cells cultured without serine as shown in B are plotted against proliferation rates of corresponding cells cultured without serine. Data points denote the different cell lines (Calu6, A549, 8988T, MIA PaCa-2, H1299, and HCT116). **(D)** Serine labeled (M+3) from U-^13^C-glucose over time in A549 and H1299 cells cultured with and without serine for 24 hours prior to U-^13^C-glucose exposure. Serine levels are normalized to internal norvaline standard and cell number measured in indicated conditions, n=3. **(E)** Absolute fold change of serine labeled (M+3) from U-^13^C-glucose in A549 and H1299 cells cultured without serine relative to serine labeled (M+3) from U-^13^C-glucose in A549 and H1299 cells cultured with serine for 24 hours after twelve minutes of U-^13^C-glucose exposure, n=3. Data shown are means ± SD. P-values were calculated by unpaired Student’s t-test, **p<0.01, ***p<0.001

Based on our finding that the NAD+/NADH ratio correlates with serine synthesis rate, we wondered whether endogenous cell NAD+/NADH ratios, which differ across cancer cells, are predictive of proliferation rate following serine deprivation. To test this, we measured how proliferation rate varied in a panel of cancer cells derived from various tissues-of-origin and with different genetic driver mutations, and found a wide range of sensitivity to serine deprivation (Figure 2A, **Supplementary Figure 2A, Supplementary Table 1**). Because PHGDH expression is important for proliferation in serine depleted conditions (DeNicola, 2015; Locasale, 2011; Maddocks, 2017; Possemato, 2011; Sullivan, 2019b), we first assessed PHGDH protein expression across the different cancer cells to examine how well this explained environmental serine dependence. As expected, some cells with low PHGDH protein expression (MCF7, MDA-MB-231) were most sensitive to serine withdrawal, and some cells with higher PHGDH protein levels were more resistant to serine withdrawal (PHGDH-overexpressing MDA-MB-231, A375) (**Supplementary Figure 2A-C**). However, for many cancer cells PHGDH expression alone could not fully explain sensitivity to serine withdrawal (**Supplementary Figure 2D**). For example, when comparing the non-small cell lung cancer cells A549 and H1299, H1299 cells express less PHGDH protein than A549 cells but are more resistant to serine deprivation **(Supplementary Figure 2B-D)**. Consistent with lower PHGDH protein expression, H1299 cells express lower levels of NRF2, a transcriptional regulator of serine biosynthesis enzymes, including PHGDH (DeNicola, 2015). Importantly, A549 and H1299 cells proliferate similarly in serine replete conditions (A549: 1.17 doublings per day; H1299: 1.33 doublings per day), arguing that differences in basal proliferation cannot explain the different sensitivities to serine depletion (**Supplementary Figure 2A**). Given the finding that the NAD+/NADH ratio correlates with serine synthesis rate, we hypothesized that differences in the NAD+/NADH ratio upon serine deprivation could contribute to the variability in sensitivity to serine withdrawal across cells with similar PHGDH protein levels (Calu6, A549, PaTu8988T, MIA PaCa-2, H1299, and HCT116) (**Supplementary Figure 2B-D**). To test this, we measured the cell NAD+/NADH ratios in the presence and absence of exogenous serine. Interestingly, we find that serine starvation elevated the NAD+/NADH ratio in some cells, while minimally changing the NAD+/NADH ratio in others (Figure 2B, **Supplementary Figure 2E**). Strikingly, cells with elevated cell NAD+/NADH ratios following serine withdrawal were more resistant to serine depletion compared to cells with unchanged NAD+/NADH ratios (Figure 2C). The NAD+/NADH ratio was also elevated in cancer cells with higher PHGDH protein levels upon serine depletion (A375, PHGDH-overexpressing MDA-MB-231) (**Supplementary Figure 2B,E**). To confirm that an elevated NAD+/NADH ratio in response to serine depletion was associated with elevated serine synthesis, we compared how serine synthesis rates changed following serine withdrawal in A549 and H1299 cells. Indeed, in serine depleted conditions, H1299 cells demonstrated a higher rate of serine synthesis, a larger increase in serine synthesis, and had higher serine levels than A549 cells, consistent with the elevated NAD+/NADH ratio in H1299 cells (Figure 2D,E; **Supplementary Figure 2F,G**). Taken together, these data suggest that in some cells, the cell NAD+/NADH ratio is responsive to serine availability and increases in the NAD+/NADH ratio correlate with increased serine synthesis and proliferation rates in serine-depleted conditions independent of PHGDH protein expression.

### Increased mitochondrial respiration is associated with an elevated NAD+/NADH ratio and increased serine synthesis

**Figure 3.**
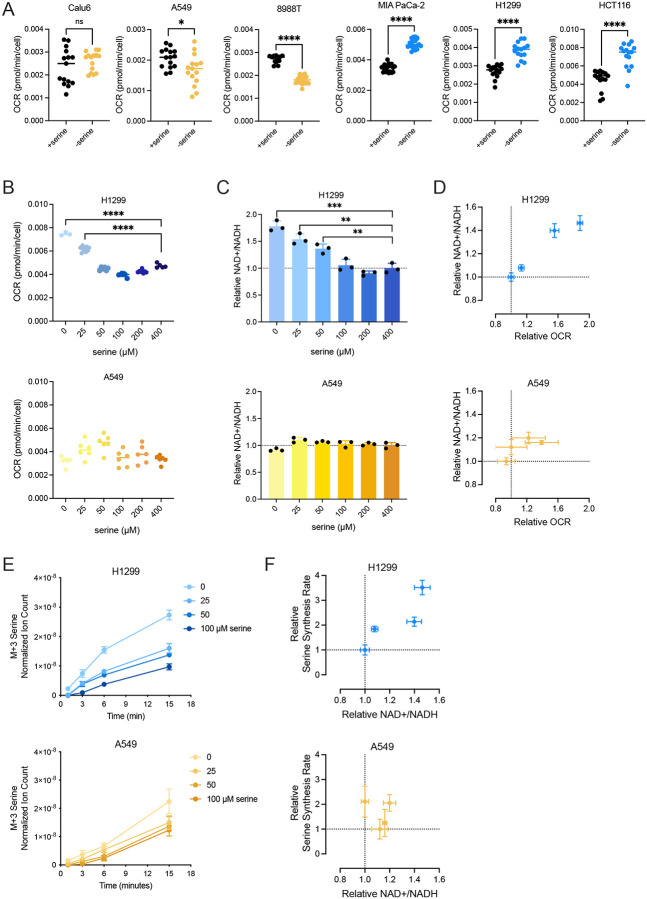
Elevated mitochondrial respiration is associated with increased NAD+/NADH ratios and serine synthesis rates. **(A)** Oxygen consumption rate (OCR) of indicated cells cultured with and without serine for 24 hours. Values are averages of three repeat measurements, n=14–20. **(B)** OCR of cells (H1299 top, A549 bottom) cultured with indicated concentrations of serine for 24 hours. Values are averages of three repeat measurements, n=3–9. **(C)** NAD+/NADH ratios of cells (H1299 top, A549 bottom) cultured with indicated concentrations of serine for 24 hours. Values are normalized to the NAD+/NADH ratio of cells cultured in 400μM serine (serine concentration in DMEM), n=3. **(D)** Relative OCR plotted against NAD+/NADH ratios in H1299 and A549 cells cultured in 0, 25, 50, and 100 μM serine for 24 hours. OCR and NAD+/NADH ratios are normalized to measurements from the 100 μM serine condition, n=3. **(E)** Serine labeled (M+3) from U-^13^C-glucose over time in cells (H1299 top, A549 bottom) cultured with indicated serine concentrations for 24 hours prior to U-^13^C-glucose exposure. Serine levels are normalized to internal norvaline standard and cell number measured in indicated conditions, n=3. **(F)** Relative NAD+/NADH ratios of cells (H1299 top, A549 bottom) cultured with 0, 25, 50, and 100 μM serine for 24 hours plotted against relative serine synthesis rates of cells cultured in corresponding conditions. Serine synthesis rates are calculated from labeled serine (M+3) after 1 and 15 minutes of U-^13^C-glucose exposure and normalized to culture conditions with serine, n=3. Data shown are means ± SD. P-values were calculated by unpaired Student’s t-test, *p<0.05, **p<0.01, ***p<0.001 ****p<0.0001, “ns” denotes not significant.

The finding that some cancer cells exhibit a more oxidized NAD+/NADH ratio upon serine starvation is surprising, particularly when increased serine synthesis will result in a higher demand for NAD+ to support increased flux through GAPDH and PHGDH (Diehl, 2019; Maddocks, 2017). We hypothesized that the elevated NAD+/NADH ratio represented a cellular response to make the NAD+/NADH ratio more oxidized to enable serine synthesis and thus sought to identify the processes that led to the increased NAD+/NADH ratio in some but not all cancer cells. Lactate production via lactate dehydrogenase (LDH) is a major NAD+ regenerating process, particularly in cancer cells with high glucose fermentation (Fantin, 2006; Luengo, 2021; Wang, 2022) (**Supplementary Figure 3A**); however, lactate secretion relative to glucose consumption was not elevated following serine deprivation in any cells tested (**Supplementary Figure 3B**). Mitochondrial respiration is another major way cells regenerate NAD+ from NADH (Handy, 2012). Thus, we tested whether changes in mitochondrial respiration, as measured by cellular oxygen consumption, accompany the increase in NAD+/NADH ratio following serine withdrawal. Indeed, cells that increased their NAD+/NADH ratio following serine deprivation also exhibited increased rates of oxygen consumption, whereas cells with unaltered NAD+/NADH ratios following serine withdrawal did not (Figure 3A, **Supplementary Figure 3C**). To test whether elevated oxygen consumption was related to serine deprivation, we cultured H1299 and A549 cells in varying amounts of extracellular serine and measured oxygen consumption rates. Oxygen consumption of A549 cells was not impacted by varying extracellular serine levels, while oxygen consumption of H1299 cells increased as extracellular serine decreased below 100 μM, the serine concentration where proliferation begins to be affected most by serine limitation (Figure 3B, **Supplementary Figure 3D**). The increase in oxygen consumption rate also corresponded with the NAD+/NADH ratio in H1299 cells at different serine levels, while the NAD+/NADH ratio in A549 cells was unchanged by extracellular serine availability (Figure 3C, D). Consistently, the NAD+/NADH ratio in H1299 cells cultured in different concentrations of serine positively correlated with serine synthesis, whereas A549 cells displayed minimal changes in serine synthesis rate (Figure 3E, F). Notably, PHGDH protein levels remained constant as extracellular serine levels were decreased in both A549 and H1299 cells (**Supplementary Figure 3E**). Together, these findings suggest that increased NAD+/NADH ratios in response to decreasing environmental serine are linked to elevated mitochondrial respiration in select cancer cells, and that this enables increased serine synthesis.

### Mitochondrial respiration governs the cell NAD+/NADH ratio and influences serine synthesis rate

**Figure 4.**
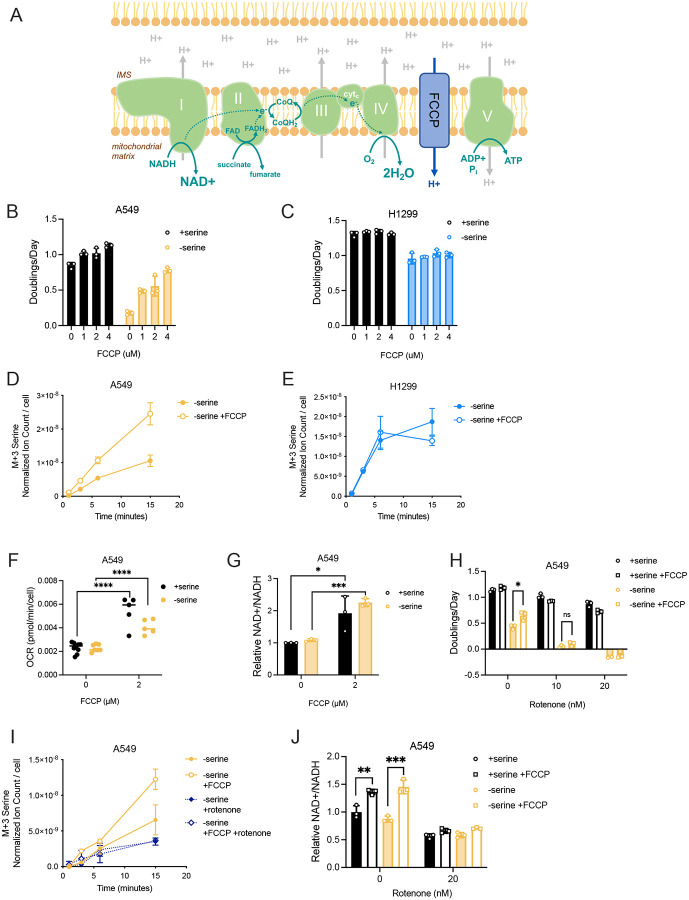
Mitochondrial respiration governs the cell NAD+/NADH ratio and influences serine synthesis. **(A)** Schematic depicting the mitochondrial electron transport chain (ETC) and the protonophore activity of FCCP that uncouples the ETC from ATP synthase activity. IMS, intermembrane space; CoQ(H_2_) indicates the oxidized and reduced (H_2_) forms of coenzyme Q; cyt_c_, cytochrome c. **(B)** Proliferation rate (doublings per day) of A549 cells cultured with and without serine with indicated concentrations of FCCP for 72 hours, n=3. **(C)** Proliferation rate (doublings per day) of H1299 cells cultured with and without serine with indicated concentrations of FCCP for 72 hours, n=3. **(D)** Serine labeled (M+3) from U-^13^C-glucose over time in A549 cells cultured without serine, and with and without 2 μM FCCP treatment for 24 hours prior to U-^13^C-glucose exposure. Serine levels are normalized to internal norvaline standard and cell number measured in indicated conditions, n=3. **(E)** Serine labeled (M+3) from U-^13^C-glucose over time in H1299 cells cultured without serine, and with and without 2 μM FCCP treatment for 24 hours prior to U-^13^C-glucose exposure. Serine levels are normalized to internal norvaline standard and cell number measured in indicated conditions, n=3. **(F)** Oxygen consumption rate (OCR) of A549 cells cultured with and without serine with indicated FCCP treatment for 24 hours. Values are averages of three repeat measurements, n=5–9. **(G)** Relative NAD+/NADH ratio of A549 cells cultured with and without serine with indicated FCCP treatment for 24 hours. NAD+/NADH ratios are normalized to the NAD+/NADH ratio in A549 cells culture with serine, n=3. **(H)** Proliferation rate (doublings per day) of A549 cells cultured with and without serine, 2 μM FCCP, and indicated concentrations of rotenone for 72 hours, n=3. **(I)** Serine labeled (M+3) from U-^13^C-glucose over time in A549 cells cultured without serine, and with and without 2 μM FCCP and 20 nM rotenone as indicated, for 24 hours prior to U-^13^C-glucose exposure. Serine levels are normalized to internal norvaline standard and cell number measured in indicated conditions, n=3. **(J)** Relative NAD+/NADH ratio of A549 cells cultured with and without serine, 2 μM FCCP, and 20 nM rotenone as indicated. NAD+/NADH ratios are normalized to the NAD+/NADH ratio in untreated A549 cells cultured with serine, n=3. Data shown are means ± SD. P-values were calculated by unpaired Student’s t-test, *p<0.05, **p<0.01, ***p<0.005, ****p<0.001, “ns” denotes not significant.

To test whether increased mitochondrial respiration following serine depletion causes an increase in the cell NAD+/NADH ratio, we treated redox unresponsive cells (i.e. cells that do not elevate oxygen consumption or the NAD+/NADH ratio following serine withdrawal) with the proton ionophore carbonyl cyanide-4-(trifluoromethoxy)phenylhydrazone (FCCP), which pharmacologically increases mitochondrial oxygen consumption by uncoupling respiration from ATP synthesis (McLaughlin, 1980; Terada, 1990). Increasing respiration with FCCP elevates mitochondrial NAD+ regeneration and the cell NAD+/NADH ratio (Luengo, 2021) (Figure 4A). Indeed, FCCP treatment dose-dependently improved proliferation of redox unresponsive cells in serine-depleted conditions (Figure 4B, **Supplementary Figure 4A**). In contrast, FCCP did not improve the proliferation of redox responsive cells grown without serine (Figure 4C, **Supplementary Figure 4B**). Consistent with its impact on proliferation, FCCP treatment led to higher serine synthesis rates only in redox unresponsive A549 cells while having no impact on the serine synthesis rates of redox responsive H1299 cells (Figure 4D,E, **Supplementary Figure 5A-D**). As expected, uncoupling mitochondrial respiration from ATP synthase with FCCP led to elevated oxygen consumption in serine depleted A549 cells (Figure 4F). FCCP also increased oxygen consumption in serine depleted H1299 cells (**Supplementary Figure 4C**). Interestingly, FCCP raised the NAD+/NADH ratios of both A549 and H1299 cells following serine withdrawal despite only improving serine synthesis and proliferation in A549 cells (Figure 4G, **Supplementary Figure 4D**). The lack of impact on serine synthesis by FCCP in H1299 cells suggests that NAD+ availability may not further constrain serine synthesis in cells that already elevate mitochondrial respiration following environmental serine depletion. In this context, we considered whether another constraint such as PHGDH protein expression was limiting serine synthesis in redox responsive cells treated with FCCP. Indeed, overexpressing PHGDH in serine-depleted redox responsive H1299 and HCT116 cells fully restored proliferation to that observed in serine-replete conditions (**Supplementary Figure 4E,G**). In contrast, proliferation of redox unresponsive cells following serine withdrawal had the greatest improvement with simultaneous PHGDH overexpression and FCCP treatment (**Supplementary Figure 4F,G**). This underscores that changes in both the NAD+/NADH ratio and relevant enzyme expression impact serine synthesis in serine depleted environments.

To investigate whether FCCP improved serine synthesis and proliferation of redox unresponsive cells through oxidizing the NAD+/NADH ratio, we treated cells with the mitochondrial complex I inhibitor rotenone in conjunction with FCCP. Rotenone treatment blocked the ability of FCCP to improve both proliferation and serine synthesis and suppressed the increase in the NAD+/NADH ratio (Figure 4H–J, **Supplementary Figure 5E,F**). In contrast, treating cells with oligomycin, an ATP synthase inhibitor, did not affect the ability of FCCP to improve proliferation (**Supplementary Figure 4H,I**). This supports the conclusion that FCCP increases serine synthesis and proliferation in serine depleted environments by increasing the NAD+/NADH ratio via mitochondrial complex I-mediated NAD+ regeneration. Moreover, FCCP treatment neither increased PHGDH protein expression nor glucose consumption, indicating that improved proliferation and serine synthesis is not caused by changes in enzyme expression or carbon availability for serine synthesis (**Supplementary Figure 4J,K**). Together, these data argue that mitochondrial respiration, either endogenously increased in response to serine deprivation or as a result of FCCP treatment, can govern the cell NAD+/NADH ratio and directly influence the serine synthesis rate in response to serine withdrawal.

### The NAD+/NADH ratio influences oxidative citrate synthesis in either serine- or lipid-depleted environments

**Figure 5.**
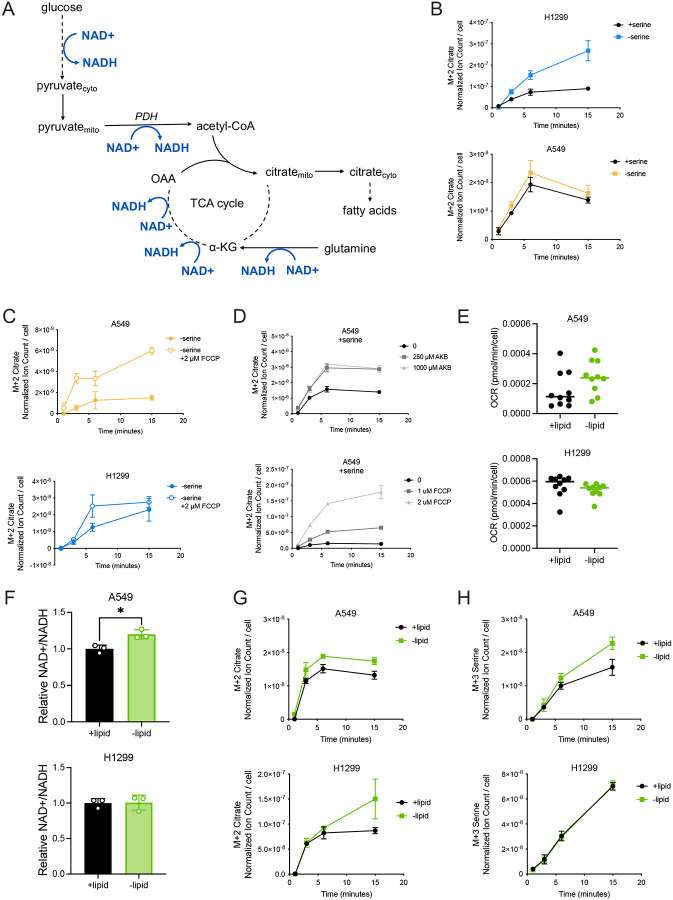
Lipid depletion leads to a cell-specific elevation in mitochondrial respiration and the NAD+/NADH ratio, influencing oxidative citrate and serine synthesis. **(A)** Schematic depicting glucose and glutamine oxidative routes for synthesizing citrate, highlighting NAD+ requiring oxidation reactions. **(B)** Citrate labeled (M+2) from U-^13^C-glucose over time in H1299 and A549 cells cultured with and without serine for 24 hours prior to U-^13^C-glucose exposure. Citrate levels are normalized to internal norvaline standard and cell number measured in indicated conditions, n=3. **(C)** Citrate labeled (M+2) from U-^13^C-glucose over time in A549 and H1299 cells cultured without serine, and with and without 2 μM FCCP for 24 hours prior to U-^13^C-glucose exposure. Citrate levels are normalized to internal norvaline standard and cell number measured in indicated conditions, n=3. **(D)** Citrate labeled (M+2) from U-^13^C-glucose over time in A549 cells cultured with serine, and with indicated concentrations of AKB or FCCP for 24 hours prior to U-^13^C-glucose exposure. Citrate levels are normalized to internal norvaline standard and cell number measured in indicated conditions, n=3. **(E)** Oxygen consumption rate (OCR) in A549 and H1299 cells cultured with and without lipids for 24 hours. Values are averages of three repeat measurements, n=10. **(F)** Relative NAD+/NADH ratios of A549 and H1299 cells cultured with and without lipids for 24 hours. NAD+/NADH ratios are normalized to the NAD+/NADH ratio of the corresponding cell type cultured with lipids, n=3. **(G)** Citrate labeled (M+2) from U-^13^C-glucose over time in A549 and H1299 cells cultured with and without lipids for 24 hours prior to U-^13^C-glucose exposure. Citrate levels are normalized to internal norvaline standard and cell number measured in indicated conditions, n=3. **(H)** Serine labeled (M+3) from U-^13^C-glucose over time in A549 and H1299 cells cultured with and without lipids for 24 hours prior to U-^13^C-glucose exposure. Serine levels are normalized to internal norvaline standard and cell number measured in indicated conditions, n=3. Data shown are means ± SD. P values were calculated by unpaired Student’s t-test, *p<0.05.

Oxidative biosynthetic reactions other than serine synthesis can be constrained by the NAD+/NADH ratio. For example, cancer cells deprived of environmental lipids increase oxidative citrate production, which can be limited by NAD+ regeneration (Li, 2022) (Figure 5A, **Supplementary Figure 6A**). Interestingly, analysis of kinetic U-^13^C-glucose tracing in cells that showed increased NAD+/NADH ratios following serine deprivation largely showed elevated M+2 citrate production despite no change in environmental lipid availability (Figure 5B; **Supplementary Figure 6B-E**). In contrast, cells with unaltered NAD+/NADH ratios upon serine depletion did not exhibit higher M+2 citrate production (Figure 5B; **Supplementary Figure 6B, F-H**). Similarly, only cells that increased the NAD+/NADH ratio in response to serine depletion displayed evidence of elevated citrate production from glutamine either through oxidative decarboxylation or reductive carboxylation, both of which require oxidation reactions that can be limited by electron acceptor availability (Hosios and Vander Heiden, 2018; Li, 2022) (**Supplementary Figure 6A, I, J**). To confirm that elevated M+2 citrate synthesis was driven by an increase in the NAD+/NADH ratio, we treated A549 cells depleted of serine with FCCP and examined glucose derived citrate synthesis. Indeed, FCCP treatment led to a four-fold increase in M+2 citrate from U-^13^C-glucose in A549 cells depleted of serine whereas M+2 citrate synthesis was less affected by FCCP in H1299 cells (Figure 5C). The elevated M+2 citrate production in FCCP treated-A549 cells was dependent on mitochondrial complex I, as rotenone prevented the elevated citrate production associated with FCCP treatment (**Supplementary Figure 7A-C**). To confirm that higher M+2 citrate production following FCCP treatment was related to the NAD+/NADH ratio independent of serine deprivation, we treated A549 cells with increasing doses of AKB and FCCP in serine replete conditions and find that increasing the NAD+/NADH ratio with either AKB or FCCP led to elevated M+2 citrate synthesis (Figure 5D). This emphasizes that changes to the NAD+/NADH ratio regardless of the nutrient environment can impact citrate synthesis.

We next asked whether increasing mitochondrial respiration in response to low extracellular serine was a general response to other nutrient deprivations that increased NAD+ demand. To examine this, we depleted A549 and H1299 cells of extracellular lipids by culturing them in media with lipid-depleted serum (Hosios, 2018) and compared oxygen consumption rates to those of cells cultured in media with lipid-containing serum. Surprisingly, we find that A549 cells, which do not increase mitochondrial respiration in response to serine depletion, mildly increase oxygen consumption rates upon lipid depletion. On the other hand, H1299 cells do not increase oxygen consumption in lipid depleted conditions (Figure 5E). We next measured the NAD+/NADH ratio of A549 and H1299 cells depleted of environmental lipids. Consistent with the changes in oxygen consumption, A549 cells displayed a slightly increased NAD+/NADH ratio while H1299 cells did not alter the NAD+/NADH ratio upon lipid deprivation (Figure 5F). Interestingly, both serine and citrate produced via glucose oxidation were elevated in lipid-deprived A549 cells in comparison to H1299 cells, which exhibited unchanged serine synthesis in response to environmental lipid depletion (Figure 5G–H, **Supplementary Figure 8A-D**). These findings demonstrate that the NAD+/NADH ratio is a determinant of both oxidative citrate and serine synthesis, regardless of environmental demand for the nutrient. Additionally, the different metabolic responses to lipid and serine deprivation between cancer cell types suggests that different nutrient environments may impact mitochondrial respiration through distinct processes to influence the cell NAD+/NADH ratio.

### Lipid depletion can enhance serine synthesis and proliferation in serine deprived conditions

**Figure 6.**
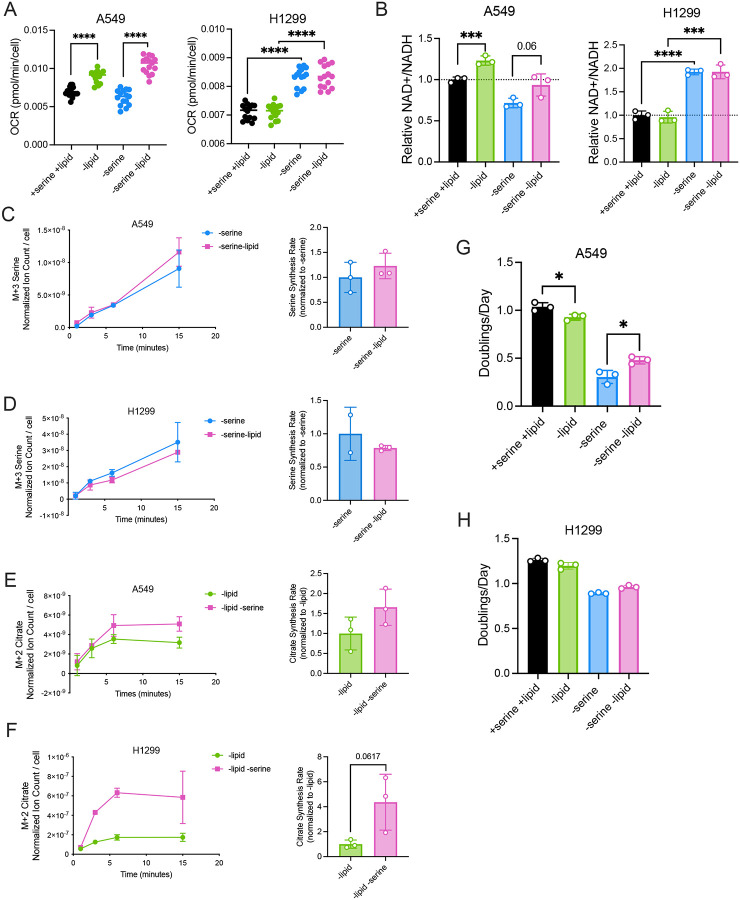
Lipid depletion increases mitochondrial respiration and the NAD+/NADH ratio in a cell specific manner to influence serine synthesis and proliferation in serine-depleted conditions. **(A)** Oxygen consumption rate (OCR) of A549 and H1299 cells cultured with and without serine or lipids for 24 hours. Values are the average of three repeat measurements, n=14–15. **(B)** Relative NAD+/NADH ratios of A549 and H1299 cells culture with and without serine or lipids for 24 hours. NAD+/NADH ratio is normalized to the NAD+/NADH ratio in corresponding cell type cultured with serine and lipids, n=3. **(C, D)** Left: Serine labeled (M+3) from U-^13^C-glucose over time in A549 cells (C) and H1299 cells (D) cultured without serine, and with or without lipids for 24 hours prior to U-^13^C-glucose exposure. Right: Serine synthesis rates of A549 cells (C) and H1299 cells (D) cultured in conditions shown on left. Serine synthesis rates are calculated from labeled serine (M+3) after 1 and 15 minutes of U-^13^C-glucose exposure and normalized to the serine synthesis rates of the corresponding cell type cultured without serine and with lipids. Serine levels are normalized to internal norvaline standard and cell number measured in indicated conditions, n=3. Single data point (H1299, -serine +lipid, t=15 min) removed due to error in sample processing. (**E,F)** Left: Citrate labeled (M+2) from U-^13^C-glucose over time in A549 cells (E) and H1299 cells (F) cultured without lipids, and with or without serine for 24 hours prior to U-^13^C-glucose exposure. Right: Citrate synthesis rates of A549 cells (E) and H1299 cells (F) cultured in conditions shown on left. Citrate synthesis rates are calculated from labeled citrate (M+2) after 1 and 15 minutes of U-^13^C-glucose exposure and normalized to the citrate synthesis rates of the corresponding cell type cultured without lipids and with serine. Citrate levels are normalized to internal norvaline standard and cell number measured in indicated conditions, n=3. **(G)** Proliferation rate (doublings per day) of A549 cells cultured with and without serine or lipids for 72 hours, n=3. **(H)** Proliferation rate (doublings per day) of H1299 cells cultured with and without serine or lipids for 72 hours, n=3. Data shown are means ± SD. P-values were calculated by unpaired Student’s t-test, *p<0.05 ***p<0.005. ****p<0.001.

Our finding that both serine and oxidative citrate synthesis are impacted by levels of a different nutrient than that produced by each pathway raised the possibility that the NAD+/NADH ratio coordinates biomass synthesis in response to the availability of multiple oxidized nutrients. Specifically, we hypothesized that changes to the NAD+/NADH ratio caused by one oxidized nutrient depletion could influence the synthesis of, and in turn the dependency on, another oxidized nutrient for proliferation. To begin testing this, we first examined how mitochondrial respiration and the NAD+/NADH ratio were impacted by dual lipid and serine depletion compared to single nutrient depletions in both A549 and H1299 cells. We find that dual depletion of serine and lipids in A549 cells led to elevated oxygen consumption that is comparable to that observed with lipid depletion alone, and higher than that observed with serine depletion alone (Figure 6A). Similarly, dual depletion of serine and lipids in H1299 cells led to elevated oxygen consumption that is comparable to that observed with serine depletion alone, and higher than that observed with lipid depletion alone (Figure 6A). The resulting oxygen consumption rates upon dual serine and lipid starvation corresponded with changes to the cell NAD+/NADH ratio (Figure 6B). We then measured the serine and citrate synthesis rates of cells depleted of both extracellular serine and lipids via kinetic U-^13^C-glucose tracing. Consistent with elevation of the NAD+/NADH ratio after lipid deprivation, A549 cells depleted of both environmental serine and lipids trended towards exhibiting a slightly elevated serine synthesis rate when compared to serine deprivation alone (Figure 6C). Moreover, lipid depletion led to a greater fraction of total serine derived from glucose in serine depleted A549 cells **(Supplementary Figure 9A)**. In contrast, lipid starvation did not alter serine synthesis in serine-depleted H1299 cells (Figure 6D, **Supplementary Figure 9B**). Interestingly, in both A549 and H1299 cells, dual serine and lipid deprivation led to elevated citrate production (to a greater degree in H1299 cells) compared to lipid deprivation alone despite only H1299 cells exhibiting a more oxidized NAD+/NADH ratio upon serine depletion (Figure 6E,F; **Supplementary Figure 9C,D**). This underscores the multifactorial regulation of glucose oxidation to citrate (Holness, 2003; Novelli, 1950; Patel, 2006; Srere, 1974). We next asked how cancer cell proliferation was impacted by dual serine and lipid depletion compared to single nutrient depletions. Strikingly, proliferation of serine-depleted A549 cells was improved with depletion of extracellular lipids (Figure 6G), consistent with an elevation in mitochondrial respiration, NAD+/NADH ratio, and serine synthesis in A549 cells upon dual serine and lipid depletion. Unlike serine depletion, lipid depletion does not cause a major proliferation defect in either A549 or H1299 cells, and dual serine and lipid deprivation did not greatly alter the proliferation of H1299 cells compared to serine depletion alone (Figure 6H). This is consistent with lipid deprivation causing no major change in oxygen consumption, NAD+/NADH ratio, and serine synthesis in H1299 cells. Taken together, these data argue that the cell NAD+/NADH ratio is modulated in response to multiple nutrient availabilities in a cancer cell-specific fashion. The changes in NAD+/NADH ratio in turn influence the capacity to synthesize oxidative biomass precursors such as serine and citrate, impacting the proliferation rate of cancer cells in nutrient environments with differing demands for oxidative biomass synthesis.

## Discussion

Proliferating cancer cells must acquire sufficient biomass and rely on contributions from both environmental biomass precursors and de novo biomass synthesis, but what determines which cells can adapt to different nutrient environments that impose constraints on salvage and de novo synthesis is not known. This study highlights that differential cell responses to change NAD+/NADH ratio enables biomass synthesis and determines whether cells can adapt to proliferate in different nutrient conditions. Importantly, this provides one explanation for why single nutrient levels in tissues may not predict the ability of auxotrophy for a nutrient to determine cancer proliferation in a tissue (Abbott, 2024). It also suggests why cancer proliferation in different nutrient environments is controlled by a complex relationship between cell-intrinsic and cell-extrinsic factors. These factors may be coordinated in part through processes that set the cell NAD+/NADH ratio.

Most prior work to determine what shapes the metabolic dependencies of cell proliferation has focused on factors that govern biosynthetic enzyme expression and activity. For example, genomic and transcriptional analyses of cancer discovered that increased PHGDH expression promotes tumor growth (DeNicola, 2015; Locasale, 2011; Newman, 2017; Possemato, 2011) and provides a proliferative advantage in serine depleted tumor microenvironments (Ngo, 2020; Sullivan, 2019b). Our findings show that enzyme expression alone cannot fully explain nutrient dependencies, as PHGDH expression can be discordant with sensitivity to serine depletion. Rather both the NAD+/NADH ratio and enzyme expression play integral roles in determining cancer proliferation in specific nutrient environments. These data are consistent with biosynthetic gene expression and single nutrient levels being unable to predict the tumor tissue environments where cancer cells can form metastases (Abbott, 2024).

Extensive work has highlighted the importance of cell NAD+/NADH ratio in influencing oxidized biomass production, nutrient utilization, and nutrient dependencies (Baksh, 2020; Bao, 2016; Birsoy, 2015; Broeks, 2023; Christensen, 2014; Diehl, 2019; Garcia-Bermudez, 2018; Gibson, 1976; Krall, 2021; Li, 2022; Schwörer, 2020; Sullivan, 2015; Wu, 2024). Yet, the processes that govern the endogenous NAD+/NADH ratio upon shifts in nutrient availability have been largely uncharacterized. We find that regulation of mitochondrial respiration is a major mechanism that dictates the NAD+/NADH ratio in response to environmental nutrient fluctuations. The mitochondrial respiration response to withdrawal of specific nutrients appears to vary across different cancers. Some cancer cells increased mitochondrial respiration to serine depletion, while others had minimal responses. On the other hand, lipid depletion yielded mitochondrial respiration changes in a different set of cells. No clear common genetic factor or tissue-of-origin was found to align with those cells that do or do not have mitochondrial and redox responses to serine and/or lipid depletion, and further work is needed to understand the mechanisms that enable increased mitochondrial respiration to respond to nutrient deprivation. One possibility is that differences in ATP consumption in response to nutrient deprivation contribute to the response. Stimulating ATP consumption can increase mitochondrial respiration and the NAD+/NADH ratio by modulating mitochondrial membrane potential (Bertholet, 2019; Buttgereit, 1995; Fisher-Wellman, 2012; Luengo, 2021; Nobes, 1989; Vander Heiden, 1999). This suggests that ATP synthase activity can constrain complex I activity to different extents across cancers, modulating the NAD+/NADH ratio and sensitivity to serine or lipid depletion. However, the regulation of complex I, ATP synthesis, and mitochondrial coupling efficiency is complicated and incompletely understood (Alavian, 2014; Gao, 2018; Lapuente-Brun, 2013; Luengo, 2021; Qiu, 2014; Wang, 2022; Yao, 2019; Zhao, 2019). Nevertheless, our findings underscore a relationship between environmental nutrient levels and mitochondrial respiration to influence the NAD+/NADH ratio in cells.

Efforts to understand the processes that shape cancer cell metabolic dependencies have largely focused on how cancer cells respond to single nutrient changes. However, through dual serine and lipid depletion experiments, these findings suggest that single nutrient availabilities will be unable to explain cancer metabolic dependencies in some contexts. For example, depleting A549 cells of both extracellular serine and lipids paradoxically improved proliferation relative to serine deprivation alone because the cell NAD+/NADH ratio responds to lipid deprivation to enable increased serine synthesis. This suggests that the NAD+/NADH ratio can serve as a metabolic regulatory node that orchestrates biomass synthesis and integrates changes to multiple oxidized biomass components in the environment, providing a framework for understanding why cancer nutrient dependencies vary across tissue microenvironments (Abbott, 2024). Because oxidizing the cell redox state can increase flux through multiple pathways, the impact of cell redox state on biosynthesis may also explain why some cells lose enzyme expression as this could redirect limited resources toward biomass components that can otherwise be scavenged from the environment (Delage, 2010; Pavlova, 2017; Rabinovich, 2015). Better understanding the mechanisms cells use to alter respiration to adjust the NAD+/NADH ratio in response to nutrient changes could lead to better understanding of the complex interplay between cell-intrinsic and cell-extrinsic factors that determine cancer metabolic dependencies and enable better selection of patients who will respond to therapies targeting metabolism.

## Materials and Methods

### Cell Culture Experiments

Cell lines were maintained in Dulbecco’s Modified Eagle’s Medium (DMEM) without sodium pyruvate (Corning 50–013-PC) supplemented with sodium bicarbonate (Sigma S6014) and 10% heat-inactivated Fetal Bovine Serum (FBS). All cells were cultured at 37°C with 5% CO_2_, tested for mycoplasma contamination regularly, and confirmed negative before experimentation. Cell line information is listed in Supplementary Table 1.

### Proliferation Assays

Cells were plated in six-well plates in 2 ml of DMEM with 10% heat-inactivated FBS at an initial seeding density of 40,000 cells. Cells were permitted to settle overnight and then quantified using a sulforhodamine B (SRB) (Sigma Aldrich 230162) colorimetric assay to measure the cell absorbance at the start of the experiment as previously described (Vichai, 2006). For treatment conditions, cells were washed three times with 1 ml of 1X PBS, and 2 ml of treatment media were added to each well. All treatment media was made with 10% dialyzed FBS. Serine-free medium was made by adding a mix of amino acids (Sigma Aldrich) at DMEM concentrations lacking serine and glycine to DMEM with low glucose, without sodium pyruvate, and no amino acids (US Biological D9800-13-50L). Glucose and glycine were supplemented for a final concentration of 25 mM and 0.4 mM, respectively. 72 hours after the initial treatment, cells were quantified using SRB assay. All SRB measurements are normalized to a blank to correct for background signal. Proliferation rates were calculated with the following formula:

$$\text{Doublings per day}=\frac{log_{2}(\text{final SRB absorbance}/\text{SRB absorbance day}\;0)}{\left(\text{total}\;\#\;\text{of treatment days}\right)}$$

### NAD+/NADH Measurements

NAD+/NADH measurements were performed using the NAD/NADH-Glo Assay (Promega G9072) with a modified version of manufacturer instructions as previously reported (Sullivan, 2015). Cells were plated at an initial seeding density of 20,000 cells (A549, H1299, HCT116) or 40,000 cells (Calu6, MDA-MB-231). Cells were permitted to settle overnight. The next day, cells were washed once with 1X PBS and then incubated with 2 ml of treatment media for the indicated times prior to preparation of cell extracts. For extraction, cells were washed once in ice cold 1X PBS and extracted in 100 μl ice-cold 1% dodecyltrimethylammonium bromide (DTAB) in 0.2 N NaOH diluted 1:1 with 1X PBS. Each sample was flash-frozen in liquid nitrogen and immediately stored at −80°C. To measure NADH, 10 μl of sample was moved to PCR tubes, diluted with 10 μl of DTAB, and incubated at 75 °C for 30 min, where basic conditions selectively degrade NAD+. To measure NAD+, 10 μl of the samples was moved to PCR tubes containing 30 μl DTAB and 20 μl 0.4 N HCl and incubated at 60°C for 15 min, where acidic conditions selectively degrade NADH. Samples were then allowed to equilibrate to room temperature and quenched by neutralizing with 20 μl 0.25 M Tris in 0.2 N HCl (for NADH) or 20 μl 0.5 M Tris base (for NAD+). Manufacturer instructions were followed thereafter to measure NAD+/NADH using a luminometer (Tecan Infinite M200Pro). A standard curve with representative samples was done with each assay to confirm that NAD+ and NADH measurements were in the linear range of detection.

### Glucose Consumption and Lactate Secretion Measurements

Cells were plated at a density of 40,000 cells per well in a six well plate. The next day, cells were washed three times with 1X PBS before 2 ml of treatment media were added to each well. In parallel, 2 ml of treatment media were added to plates without cells. An initial cell number before beginning treatment was quantified via Cellometer (Nexcelom Bioscience). 72 hours later, media was collected from exponentially proliferating cells with a parallel collection of media from wells without cells, which we used to measure initial media concentrations to take into consideration evaporative changes. Cell number was quantified via Cellometer along with the volume of media on cells to consider evaporative changes to metabolite concentrations that may have occurred over 72 hours in culture. Glucose concentrations from media samples were measured on a YSI-2900 Biochemistry Analyzer (Yellow Spring Instruments). Every assay included a glucose (Sigma Aldrich G8270) standard curve (0, 2.5, 5, 10, 20, and 40 mM glucose) and lactate (Sigma Aldrich 71716) standard curve (0, 1.25, 2.5, 5, 10, and 20 mM sodium lactate), made in both control and treatment media. To calculate consumption rate per cell for a given nutrient, fmol of glucose or lactate over time were plotted relative to the area under the curve (fmol / cells · time) of an exponential function fit to the number of cells at the start of the assay and after 72 hours of treatment, as previously described (Hosios, 2016).

### Oxygen Consumption Measurements

An Agilent Seahorse Bioscience Extracellular Flux Analyzer (XFe96) was used to measure oxygen consumption rates (OCR). Cells were plated at 20,000 – 40,000 cells per well in a Seahorse Bioscience 96-well plate in 80 μl of DMEM without pyruvate supplemented with 10% heat-inactivated FBS. Cells were not plated on the perimeter of the plate to avoid edge effect. The following day, cells were washed twice with 180 μl of treatment media supplemented with 10% dialyzed FBS before incubating cells with 180 μl of treatment media for the indicated time prior to OCR data acquisition. For 24 hour treatment periods, treatment media was replaced with 180 μl of fresh treatment media two hours before OCR data acquisition. The day before OCR data acquisition, the XFe96 cartridge was hydrated by submerging the sensor cartridge in 200 μl of sterile water per well of the utility plate. Parafilm was wrapped around the perimeter of the cartridge to avoid evaporation. The XFe96 cartridge was then incubated in a 37°C non-CO_2_ incubator overnight. The next day, at least two hours before data acquisition, the water in the utility plate was replaced with 200 μl of the Agilent XF Calibrant per well and the sensory cartridge was submerged and returned to the 37°C non-CO_2_ incubator until OCR was ready to be measured. After OCR data acquisition, six to eight wells per treatment condition were collected for cell number quantification (Cellometer) to normalize OCR values. Basal OCR was calculated by subtracting residual OCR, the OCR remaining following the addition of rotenone and antimycin A for a final concentration of 2 μM each.

### Kinetic U-^13^C-Glucose Isotope Tracing Experiments

Cells were seeded at 150,000 cells per well in a six-well plate in 2 ml of DMEM without sodium pyruvate supplemented with 10% heat-inactivated FBS. The following day, cells were washed once with 1 ml of 1X PBS and then incubated in 2 ml of treatment media supplemented with 10% dialyzed FBS for the indicated treatment time and when metabolic steady state is reached. Two hours before the start of the kinetic isotope tracing, media on cells was exchanged with fresh treatment media with 10 mM unlabeled glucose. To measure serine labeling over time in serine-depleted conditions, cells and media were extracted together. Before the initiation of the kinetic U-^13^C-glucose isotope tracing, serine-depleted cells were washed three times with 1X PBS and then cultured with 400 μl of tracing medium containing 10 mM of U-^13^C-glucose (Cambridge Isotope Laboratories, CLM-481–0) for the rapid time points. Tracing media was equivalent to treatment media except with 10 mM of U-^13^C- glucose. Following incubation, 1.6 ml of extraction buffer containing ice cold 100% HPLC-grade methanol (Sigma Aldrich, 646377) with norvaline (Sigma, N7627) was added onto cells with tracing media for a final sample volume of 2 ml (80% extraction buffer and 20% tracing media) and a final concentration of 1 μg per 400 μl norvaline. 1.6 ml of the lysate was placed into a fresh Eppendorf tube, vortexed at 4°C at maximum speed for ten minutes, and then spun down at 4°C at maximum speed for thirty minutes. The supernatant was collected and dried under nitrogen gas to prepare for metabolic analysis. To measure serine and citrate production in serine replete conditions, only intracellular metabolites were quantified. Before the initiation of kinetic U-^13^C-glucose isotope tracing, serine-replete cells were washed three times with 1X PBS and then cultured with 1 ml of tracing medium containing 10 mM of U-^13^C-glucose (Cambridge Isotope Laboratories, CLM-481–0) for the rapid time points. Tracing media was equivalent to treatment media except with 10 mM of U-^13^C- glucose. Following incubation, cells were rapidly washed twice with ice cold blood bank saline. To extract, 500 μl of extraction buffer containing 80% ice cold HPLC-grade methanol and 20% LC-grade water spiked with 1 μg per 400 μl norvaline were added onto cells. Cells were then lysed as previously described above.

### Gas Chromatography-Mass Spectrometry (GC-MS) Polar Metabolite Measurements

Dried samples were derivatized by adding 16 μl of methoxamine reagent (Thermo Fisher, TS-45950) and incubated for an hour at 37°C followed by the addition of 20 μl of *N*-*tert*-butyldimethylsilyl-*N*-methyltrifluoroacetamide with 1% *tert*-butyldimethylchlorosilane (Sigma 375934). Samples were then incubated for two hours at 60°C. Afterwards, samples were centrifuged at maximum speed for ten minutes, and 20 μl of supernatant was used for analysis. Following derivatization, samples were analyzed using a DB-35MS column (30 m × 0.25 mm i.d. × 0.25 μm, Agilent J&W Scientific) in an Agilent 7890 gas chromatograph coupled to an Agilent 5975C mass spectrometer (GC–MS). All ion counts were normalized to internal standard norvaline and cell number measured via Cellometer.

### Immunoblotting

Cells were washed with ice cold 1X PBS and lysed in cold RIPA buffer containing Halt Protease and Phosphatase Inhibitor Cocktail (Thermo Scientific 78442). Lysates were clarified by rocking samples for thirty minutes at 4°C and then spun at maximum speed for ten minutes for collection of supernatant. Protein concentration was calculating using the BCA Protein Assay (Pierce, 23225) with BSA as a standard. Lysates were resolved by SDS-PAGE using NuPAGE 4–12% Bis-Tris Protein Gels and run at 100 V. Proteins were transferred onto nitrocellulose membranes via wet transfer at 100 V. Membranes were blocked in 5% BSA in 1x TBST before incubating membranes with primary antibodies at 4°C overnight. The next day, membranes were washed three times with 1x TBST at room temperature and then incubated in secondary antibodies for one hour at room temperature. The primary antibodies used were PHGDH (Sigma Aldrich, HPA021241 [1:1000]), HSP90 (Cell Signaling Technology, 4874 [1:1000]), and Vinculin (E1E9V) XP^®^ (Cell Signaling Technology, 13901 [1:1000]). The secondary antibodies used were anti-rabbit IgG horseradish peroxidase-linked antibody (1:4000 dilution; Cell Signaling Technologies, 7074S) or anti-mouse IgG horseradish peroxidase-linked antibody (1:4000, 7076S).

### Cell Line Generation

Cell lines overexpressing either EGFP or human PHGDH were generated via lentiviral infection. pLJM1-EGFP was obtained from Addgene (Addgene plasmid # 19319). pLJM1-PHGDH was constructed using pLJM1-Empty (Addgene plasmid # 91980) as a backbone. pLJM1-Empty was digested using NheI and EcoRI and gel purified. Human PHGDH cDNA was amplified from pLHCX-PHGDH (PMID: 31598584) with the following oligonucleotide primers, where the capitalized sequence denotes PHGDH homology:

PHGDH F: agtgaaccgtcagatccggctagcgccaccATGGCTTTTGCAAATCTGCG
PHGDH R: tactgccatttgtctcgaggtcgagaattcTTAGAAGTGGAACTGGAAGGCT

PHGDH insert was gradient PCR amplified using Phusion High-Fidelity DNA polymerase (NEB M0530) from 60 to 70°C and gel purified. Digested pLJM1-Empty and amplified PHGDH insert were assembled via Gibson Assembly, and the resulting plasmid was transformed, and validated by Sanger sequencing. Lentiviral production was done by transfecting constructs into LentiX293T cells with Mirus Transit293T (Mirus Bio MIR2700) following manufacturer’s protocol. Briefly, 800,000 cells were plated per well in a 6 well plate in DMEM media with 10% heat-inactivated FBS. The next day, cells were transfected with 1.6 μg vector (either pLJM1-EGFP or pLJM1-PHGDH), 800 ng of pMDLg (Addgene plasmid # 12251) packaging plasmid, 400 ng of pMD2.G (Addgene plasmid # 12259) envelope plasmid, and 400 ng of pRSV-REV (Addgene plasmid # 12253) in OptiMEM (Thermo Fisher Scientific 31985070). DNA transfection mix was incubated with cells for 48 hours before harvesting virus and filtering through a 0.45 μm polyethersulfone membrane. Sub-confluent cells were infected with 1 ml of virus-containing media in each well of a 6 well plate with polybrene (8 μg/ml, EMD Millipore TR-1003-G). 24 hours after infection, selection with 1 μg/mL puromycin (Sigma Aldrich P7255) began. All experiments with generated cell lines were conducted on a polyclonal cell population.

### Statistics and Reproducibility

All statistical tests using experimental data were performed with Prism 10 software. No statistical method was used to predetermine sample sizes. Samples sizes were chosen based on pilot experiments using three or more technical replicates. Certain data points were excluded from Seahorse analyzer assays due to premature injection of rotenone and antimycin. The experiments were not randomized and the investigators were not blinded during experiments nor data analysis.
